# Expanding Diversity of Susceptible Hosts in Peste Des Petits Ruminants Virus Infection and Its Potential Mechanism Beyond

**DOI:** 10.3389/fvets.2020.00066

**Published:** 2020-02-28

**Authors:** Yongxi Dou, Zhongxiang Liang, Meera Prajapati, Rui Zhang, Yanmin Li, Zhidong Zhang

**Affiliations:** ^1^State Key Laboratory of Veterinary Etiological Biology, Lanzhou Veterinary Research Institute, Chinese Academy of Agriculture Sciences, Lanzhou, China; ^2^CAAS-ILRI Joint Laboratory for Ruminant Disease Control, Lanzhou Veterinary Research Institute, Chinese Academy of Agriculture Sciences, Lanzhou, China; ^3^Animal Health Research Division, Nepal Agricultural Research Council, Lalitpur, Nepal; ^4^College of Life Science and Technology, Southwest Minzu University, Chengdu, China

**Keywords:** *Morbillivirus*, peste des petits ruminants, peste des petits ruminants virus, susceptible hosts, expanding, potential mechanism

## Abstract

Peste des petits ruminants (PPR) is a severe respiratory and digestive tract disease of domestic small ruminants caused by PPR virus (PPRV) of the genus *Morbillivirus*. Although the primary hosts of PPRV are goats and sheep, the host range of PPRV has been continuously expanding and reported to infect various animal hosts over the last decades, which could bring a potential challenge to effectively control and eradicate PPR globally. In this review, we focused on current knowledge about host expansion and interspecies infection of PPRV and discussed the potential mechanisms involved.

## Introduction

Peste des petits ruminants (PPR) is a highly contagious fatal viral disease of small ruminants characterized by fever, pneumonia, diarrhea, and inflammation of the respiratory and digestive tracts. The morbidity and mortality rates of PPR can reach up to 100%. Therefore, it has a severe socio-economic impact in the livestock industry in countries whose economy relies on small ruminants, particularly in endemic poor countries. After the successful global eradication of Rinderpest (RP) in 2011, the Food and Agriculture Organization (FAO) and World Organization for Animal Health (OIE) have targeted PPR as the next aim for its global eradication. The etiological agent PPR virus (PPRV) is a member of the genus *Morbillivirus*, family *Paramyxoviridae* and order *Mononegavirales*. PPRV primarily infects goats and sheep, but over the last decades the host range of PPRV has been continuously expanding to many other non-natural hosts by unknown mechanisms.This indicates that PPRV has a potential capability of adapting to various new hosts, which might impact on the successful implementation of the PPR global eradication plan. In this review, recent epidemiological findings of PPRV are summarized based on transmission and evolution in relation to PPRV host expansion and interspecies infection, and the potential mechanism beyond was then discussed.

## Global Distribution of Peste Des Petits Ruminants Virus

### Peste Des Petits Ruminants Virus Spreads Alarmingly Over Last Decades

**Figure 1:**
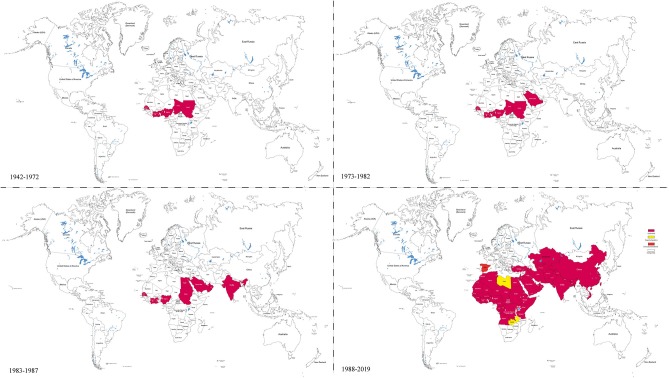
Global distribution of PPRV at different time periods. This figure is drawn according to the relevant reports of PPR and the **Table 1** references by Illustrator CS6 software. It shows that the PPRV spread from west to east and the infection is rapidly expanding.

Since its first report in 1942 in Cote-d'Ivoire, PPR has spread far beyond its origin in Western Africa (Figure 1). In 1999, the prevalence of PPRV antibodies was reported to be 29.2 and 20% in sheep and goats in Turkey/Europe, respectively (18). PPRV reemerged in many African countries including Tanzania (2008 & 2013) (7, 39), Kenya (2014) (40), Democratic Republic of Congo and Angola (2012) (41), and in North Africa such as in Tunisia (2012–2013), Morocco (2015), Algeria (2014) (42–44), and Burundi (2017) (45). In Asia, the virus spread to China in 2007 and again in 2013, spreading rapidly throughout 22 provinces (46–48), and from 2013 to 2014, PPR was also reported from countries surrounding China such as India, Vietnam, and Pakistan where high level of antibody to PPRV was observed in small domestic ruminants and wildlife (49). A risk assessment of PPRV infection in developing countries indicated that ~63% of small ruminants were at risk of infection (50). Therefore, over the last two decades, PPR dissemination has increased exponentially. According to OIE data, PPR was reported in 39 countries in 2007, 43 countries in 2013, and is present in over 70 countries across Asia, Africa, and Europe (Figure 1). As a result, PPR affects 30 million small ruminants yearly, resulting in the economic loss of approximately US$1.2–1.7 billion (42, 43, 51–53). Therefore, following the global eradication of Rinderpest (RP) in 2011, which is the first animal disease and the second disease to have been eradicated in the world, the World Organization for Animal Health (OIE) and Food and Agriculture Organization (FAO) have identified PPR as the next target for eradication by 2030.

### Peste Des Petits Ruminants Virus Origin and Evolutionary Relationship Among *Morbilliviruses*

As a member of *Morbilliviruses*, PPRV is a negative-sense, single-stranded RNA virus of ~16,000 nucleotides (nt), which consists of six open reading frames encoding for six structural proteins: Nucleocapsid (N), Phosphoprotein (P), Matrix (M), Fusion (F), Hemagglutinin-neuraminidase (HN), Large RNA-dependent polymerase (L), and two non-structural protein (C and V) (27). The HN and F proteins are embedded in the viral envelope, which constitute the fibers of the virion surface. Only one serotype of PPRV is so far known. However, phylogenetic analysis based on the small region of the N/F gene classifies PPRV into four distinct lineages (I–IV) (41, 54). In comparison to the phylogenetic analysis based on the N, F, M, and HN genes, HN gene seems to be more important to evaluate the epidemiology and the circulating PPRV in endemic areas (55), which may be due to the fact that HN protein is a major determinant for the host tropism. For many years PPR was considered as a variant of RP, specifically adapted for goats and sheep and have lost its virulence for cattle. However, it is now revealed that the PPRV and RP virus (PRV) are biologically and epidemiologically distinct although they are closely related antigenically.

**Figure 2:**
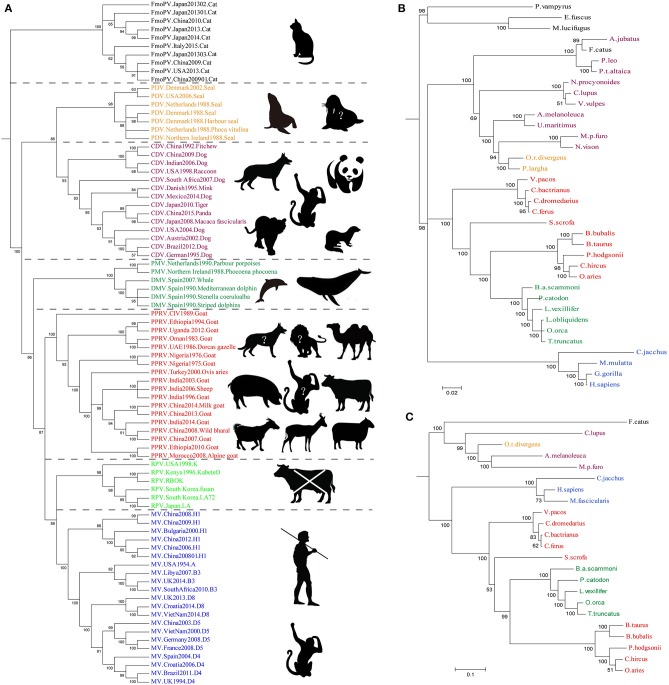
The Bayesian Phylogenetic tree was constructed based on *Morbillivirus* Hemagglutinin and its receptors SLAM and Nectin-4 gene. **(A)** The genetic relationship of MV and PPRV was the closest of morbilliviruses, and PDV and CDV were located on the same evolutionary branch. The genetic relationship of FmoPV and the other morbilliviruses was highly distant. In the right side of the tree, the host or suspicious host exhibits a broad host range of the viruses. The animals tagged with “?” represent potentially infected hosts. **(B,C)** Phylogenetic tree of SLAM and Nectin-4. The evolutionary tree shows that the evolution of the H seems to be strongly associated with the evolution of the SLAM and Nectin-4 receptor, especially in the Nectin-4. The sequences which are used to construct the Bayesian Phylogenetic tree GenBank accession numbers are listed in Table 2.

Until 1988, the genus *Morbillivirus* was thought to comprise only four viruses, namely, Measles virus (MV), PPRV, RPV, and Canine distemper virus (CDV) (56). Later on, three new morbilliviruses have been identified, namely, cetacean morbillivirus (CeMV), phocine distemper virus (PDV) (57–60), and feline morbillivirus (FmoPV or FMV) (61). Previously, clinical description and analysis of the genetic relationship suggested that MV may have originated from an ancestral morbillivirus, and RPV was believed to be the most ancient of morbilliviruses (62–65). Phylogenetic tree of morbilliviruses based on H gene indicated that the genetic relationship of MV and PPRV was the closest of morbilliviruses, and phocine distemper virus (PDV) and CDV were located on the same evolutionary branch (Figure 2). However, the genetic relationship of FmoPV and the other morbilliviruses was highly distant. Molecular evolution analysis of the viruses suggested that the time to the most recent common ancestors (TMRCA) of PPRV/MV/RPV first appeared during 1616 [95% HPD (highest posterior density) 1072–1859] (66). A TMRCA of PPRV was estimated to be in 1904 (95% HPD 1730–1966). Considering the prediction of TMRCA as reasonable and referring to the evolutionary origin theory of measle viruses, it is also possible to estimate that PPRV may have evolved from the ancient virus (RPV). Although, the first description of PPRV was documented in 1942, it was revealed as a new member of the genus *Morbillivirus* based on the biological and biochemical characteristics in 1979 (67). A study on the phylogenetic analysis of PPRV HN gene (68) exhibited that PPRV strain which caused an outbreak in China in 2007 was not the ancestor virus which caused an outbreak in China in 2013–2014. Around the twenty-first century, genetic diversity of PPRV dramatically increased and caused many outbreaks of PPR as described above. The reasons for this phenomenon are not fully understood, but this could be due to the impact of the RPV eradication program which may promote PPRV to spread rapidly.

## Cross-Species Infection of Peste Des Petits Ruminants Virus

### Expanding Diversity of Susceptible Hosts

PPRV primarily infects domestic goats and sheep, in which mortality can reach up to 100%. However, there are increasing reports of PPRV infection in other domestic and wild animals with or without showing clinical symptoms (Table 1).

**Table 1:** PPR infection cases in diverse animals except domestic small ruminants.

**Name**	**Latin name**	**Country**	**Time sampling**	**Positivity**	**References**
Buffaloes	*Bubalus bubalis*	Cote d'Ivoire Pakistan India Tanzania	2005 2006 2009 1995 2009–2010 2011 2014	- 60/89 34/240 50/385 48/1001 67/432 5/10	(1) (2) (3) (4) (5) (6) (7)
Camelids	*Camelus dromedarius*	Ethiopia Nigeria Sudan Iran Kenya	2001 2011–2013 2000–2009 2000–2012 2002 2004 2008-2012 2013 2016	10/628 51/1516 38/49 214/474 14/100 - 41/1988 - 1/25	(8) (9) (10) (11) (12) (13) (14) (15) (16)
Cattle	*Bos primigenius taurus*	Ethiopia Pakistan India Tanzania Turkey Sudan	2001 2006 2009 2009–2010 2011 2011 1999–2000 2009 2002 2008–2012	46/910 18/43 24/240 60/1158 67/605 46/266 3/321 22/122 4/35 387/1501	(8) (2) (3) (5) (6) (17) (18) (19) (12) (14)
Argali	*Ovis ammon*	China	2013–2016	-	(20)
Afghan Markhor goat	*Capra falconeri*	UAE	2008–2009	-	(21)
Arabian gazelles	*Gazella gazella*	UAE	2008–2009	-	(21)
Arabian mountain gazelles	*Gazella gazella cora*	UAE	2008–2009	-	(21)
Bharals	*Pseudois nayaur*	China	2007–2008	3/4	(22)
Barbary sheep	*Ammotragus lervia*	UAE	2008–2009	-	(21)
Bushbucks	*Tragelaphus scriptus*	UAE	2008–2009	-	(21)
Capra ibex	*Capra ibex sibirica*	China	2013–2016	-	(20)
Dorcas gazelles	*Gazella dorcas*	Saudi Arabia Sudan	2002 2016–2017	138/230 8/11	(23) (24)
Goitered gazelle	*Gazella subgutturosa*	China Tanzania Turkey Sudan	2013–2016 2014 - 2008–2012	- 20/30 10/82 5/23	(20) (7) (25) (14)
Ibex	*Capra ibex*	China	2015	-	(26, 27)
Impala	*Aepyceros melampus*	Tanzania UAE	2014 2008–2009	3/3 -	(7) (21)
Nubian ibex	*Capra nubiana*	UAE	2008–2009	-	(21)
Rheem gazelles	*Gazella subgutturosa marica*	UAE	2008–2009	-	(21)
Sindh Ibex	*Capra aegagrus blythi*	Pakistan	2009	13/20	(28)
Springbuck	*Antidorcas marsupialis*	UAE	2008–2009	-	(21)
Thomson's gazelles	*Gazella thomsoni*	Saudi Arabia	2002	5/5	(23)
Wildebeest	*Connochaetes gnou*	Tanzania	2014	1/2	(7)
White-tailed deer	*Docoileus virginianus*	USA	1979	Ex	(29)
Wild goat	*Capra aegagrus*	Iraq Iran	2010–2011 2014 2015 2016	3/4 - - -	(30) (31)
Wild sheep	*Ovis orientalis*	Iran	2001 2011 2015	- - -	(31)
Water deer	*Hydropotes inermis*	China	2016	-	(32)
Lion	*Panthera leo persica*	India	-	-	(33)
Dog	*Canis familiaris*	India	2015	3/12	(34)
Pig	*Sus scrofa*	UK		Ex	(35) (36)
Mice	*Mus musculus*	UK	2000	Ex	(37)
Midges	*Culicoides imicola*	Turkey	2016	7/12	(38)

“*-” represents not mentioned and Ex represents experimentally infected*.

**Table 2:** Morbillivirus Hemagglutinin and its receptors gene sequences used for comparison study.

**Name of *Morbillivirus* HN gene sequence**		**GenBank accession numbers**	
**Feline morbillivirus(FmoPV)**			
FmoPV.Japan2013 (01,02,03).Cat		AB924122, (AB910311, AB924120, AB924121)	
FmoPV.Japan2014.Cat		AB910310	
FmoPV.China2009 (01).Cat		JQ411014, (JQ411015)	
FmoPV.China2010.Cat		JQ411016	
FmoPV.USA2013.Cat		KR014147	
FmoPV.Italy2015.Cat		KT825132	
**Phocine distemper virus (PDV)**			
PDV. Denmark2002. Seal		AF479274	
PDV.USA2006.Seal		HQ007902	
PDV.Netherlands1988.Seal		KC802221	
PDV.Denmark1988. Seal		Z36979	
PDV.Denmark1988. Harbor seal		FJ648456	
PDV.Netherlands1988. Phoca vitulina		AJ224707	
PDV.Northern Ireland1988.Seal		D10371	
**Canine distemper virus (CDV)**			
CDV.Austria2002.Dog		GQ214378	
CDV.Brazil2012.Dog		KT429765	
CDV.China1992.Fitchew		KM926612	
CDV.China2009.Dog		HQ403645	
CDV.China2015.Panda		KP677502	
CDV.Danish1995.Mink		Z47759	
CDV.German1995.Dog		X85000	
CDV.Indian2006.Dog		AM903376	
CDV.Japan2008.Macaca fascicularis		AB687721	
CDV.Japan2010.Tiger		AB619774	
CDV.Mexico2014.Dog		KT266736	
CDV.South Africa2007.Dog		FJ461717	
CDV.USA1998.Raccoon		AY548111	
CDV.USA2004.Dog		EU716337	
**Cetacean morbillivirus**			
DMV.Spain1990.Mediterranean dolphin		AJ224705	
DMV.Spain1990.Striped dolphin		AY586536	
DMV.Spain1990.Stenella coeruleoalba		HQ829973	
DMV.Spain2007.Whale		HQ829972	
PMV.Netherlands1990.Parbour porpoises		AY586537	
PMV.Northern Ireland1988. Phocoena phocoena		FJ648457	
**Peste des petits ruminants virus (PPRV)**			
PPRV.China2008.Wild bharal		JX217850	
PPRV.China2007.Goat		JF939201	
PPRV.Ethiopia2010.Goat		KJ867541	
PPRV.Ethiopia1994.Goat		KJ867540	
PPRV.Oman1983.Goat		KJ867544	
PPRV.CIV1989.Goat		EU267273	
PPRV.Nigeria1976.Goat		EU267274	
PPRV.Nigeria1975.Goat		X74443	
PPRV.Uganda2012.Goat		KJ867543	
PPRV.UAE1986.Dorcas gazelle		KJ867545	
PPRV.Turkey2000.Ovis aries		NC006383	
PPRV.Morocco2008. Alpine goat		KC594074	
PPRV.China2014.Milk goat		KP260624	
PPRV.China2013. Goat		KM091959	
PPRV.India2003.Goat		FJ750563	
PPRV.India2006.Sheep		EU344744	
PPRV.India2014.Goat		KR261605	
PPRV.India1996.Goat		GQ452016	
**Rinderpest virus (RPV)**			
RPV.South Korea.fusan		AB547189	
RPV.Kenya1996.KabeteO		X98291	
RPV.USA1998.K		Y18816	
RPV.South Korea.LA72		JN234008	
RPV.RBOK		Z30697	
RPV.Japan.LA		M17434	
**Measles virus (MV)**			
MV.Bulgaria2000.H1		FJ808736	
MV.Brazil2011.D4		KC291546	
MV.China2003.D5		EU914221	
MV.China2006.H1		JN997514	
MV.China2008(01).H1		JN997523 (JN997516)	
MV.China2009.H1		JN997527	
MV.China2012.H1		KJ136543	
MV.Croatia2006.D4		JX126962	
MV.Croatia2014.D8		KT337320	
MV.France2008.D5		GQ428197	
MV.Germany2008.D5		GQ121274	
MV.Libya2007.B3		FN594772	
MV.SouthAfrica2010.B3		KC305668	
MV.Spain2004.D4		FJ869874	
MV.UK1994.D4		GQ331933	
MV.UK2013.D8		KT732260	
MV.UK2014.B3		KT732223	
MV.USA1954.A		JX436452	
MV.VietNam2000.D5		JF728849	
MV.VietNam2014.D8		AB968381	
**Name of SLAM gene sequence**	**GenBank accession numbers**	**Name of Nectin-4 gene sequence**	**GenBank accession numbers**
*A.jubatus*	XM027048539	*A. melanoleuca*	XM002928747
*A.melanoleuca*	XM002928437	*B.a. scammoni*	XM007171734
*B.a.scammoni*	XM007171753	*B. Taurus*	NM001024494
*B.bubalis*	DQ228868	*B.bubalis*	BC148055
*B.taurus*	BC114833	*C. jacchus*	XM003735162
*C.bactrianus*	XM010955648	*C. bactrianus*	XM010955671
*C.dromedarius*	XM010993089	*C. dromedarius*	XM010993067
*C.ferus*	XM014562035	*C. ferus*	XM006173954
*C.hircus*	DQ228869.1	*C. lupus*	NM001313853
*C.jacchus*	XM002760176	*C. hircus*	MG870289
*C.lupus*	MG870622	*F. catus*	XM019822297
*E.fuscus*	XM028129350	*H. sapiens*	NM030916
*F.catus*	NM001278826	*L. vexillifer*	XM007467114
*G.gorilla*	XM004027719	*M. fascicularis*	AB742522
*H.sapiens*	NM003037	*M.p. furo*	XM004775900
*L.obliquidens*	AB428366	*O.r.divergens*	XM004407894
*L.vexillifer*	XM007467101	*O.orca*	XM004284416
*M.lucifugus*	XM014462802	*O.aries*	XM004002680
*M.mulatta*	XM001117605	*P.hodgsonii*	XM012184689
*M.p.furo*	XM004775878	*P.catodon*	XM007112295
*N.procyonoides*	EU678639	*S.scrofa*	AK397273
*N.vison*	FJ626692	*T.truncatus*	XM030842125
*O.aries*	DQ228866	*V.pacos*	XM006215669
*O.orca*	NM001279809		
*O.r.divergens*	XM004407883		
*P.catodon*	XM007124057		
*P.hodgsonii*	NM001040288		
*P.largha*	AB428368		
*P.leo*	XM019433334		
*P.t.altaica*	XM007092374		
*P.vampyrus*	XM011373055		
*S.scrofa*	AK391518		
*T.truncatus*	XM004327846		
*U.maritimus*	XM008700683		
*V.pacos*	XM015249006		
*V.vulpes*	EU678638		

#### PPR Infection in Cattle and Buffaloes

PPRV infection was also reported from cattle and buffaloes. A serological survey of 2,159 bovine samples from Southern Peninsular India between 2009 and 2010 showed the prevalence of PPRV antibody to be 5.21 and 4.82% in cattle and buffaloes, respectively (5). Further analysis of 1,498 samples collected in 2011 indicated the overall seroprevalence of 21.83% with 16.20% in buffaloes and 11.07% in cattle (6), while a high seroprevalence of 41.86% in cattle and 67.42% in buffaloes was reported from Pakistan (2). Recent serological survey detected PPRV antibodies at the rate of 17.5 and 22.5% in cattle and buffaloes, respectively (3). Additionally PPRV infection in Buffaloes (*Syncerus caffer)* was also reported in Tamil Nadu state, India (4). Experimental study showed that calves infected with PPRV developed subclinical signs of PPRV infection along with specific anti-PPRV antibodies (69). PPRV can be isolated from Peripheral blood mononuclear cell (PBMC), but the virus was not detected from oral, nasal, and rectal swabs by ELISA/RT-PCR assays (69), indicating that cattle is unlikely to pose a risk in transmitting PPRV to other animals. Nevertheless, the persistence of PPRV in infected calves for long periods is still unknown (69). Also, this study emphasizes on the importance of an investigation as to whether goat or sheep maintain PPRV for long periods subclinically. A recent study (70) also reported that the cattle infected with wild-type PPRV from each lineage (Lineage 1 strain CIV89, lineage strain 2 Nigeria 75/3; lineage 3 strain Ethiopia, and lineage 4 strain India-Calcutta) neither showed any sign of PPRV replication in the epithelial cells nor the transmission of PPR virus to in-contact animals such as goats. Similarly, a higher seroprevalence of 42% (420/1000) among cattle populations in the Sudan was reported when tested by competitive ELISA (71). Even though the infected cattle did produce specific anti-PPRV antibodies as in the previous studies mentioned above (69), cattle are unlikely to act as a PPRV reservoir and to play a role in the maintenance and transmission of PPRV because these findings indicated that cattle are a dead-end host for PPRV.

#### PPRV Infection in Camelids

There have been several reports of PPRV infection in camels. Serological survey undertaken earlier in 1995 after the occurrence of a rinder-pest-like disease syndrome in the camel population in Ethiopia showed a global seroprevalence of 7.8% for PPRV antibodies in camels (72), indicating the occurrence of PPRV infection in camels. Subsequent serological survey on naïve camels and other ruminants in Ethiopia in 2001 showed that the prevalence of the PPRV antibody to be 3% in camels, which was less compared to cattle (9%), goats (9%), and sheep (13%) (8). In addition, PPRV-positive serology in camels has been documented in other countries, with prevalence of PPRV antibody varying from 14/100 (12), 38/49 (10), 214/474 (11), and 51/1517 in Nigeria (9). A recent serological survey detected PPRV antibodies at the rate of 41/1988 (14). In addition to detection of antibodies in camels, an outbreak of PPR in camels with 7.4% mortality was reported in Sudan in mid-August 2004 (13), indicating camels may be susceptible to PPRV under field conditions. While investigating the prevalence of PPR in camels in different areas of Sudan, PPRV antigen was detected in 45.1% (214/474) of the tested pneumonic lung specimens of clinically healthy camels using immunocapture ELISA (11). Further investigation on PPR in domestic ruminants of Sudan from 2008 to 2012 showed that PPR antigen was detected in 33.6% of the lung tissue samples of camels (*n* = 1,276), which was higher than that in goats (21.1%), sheep (15.4%), and cattle (12.3%) (14). A number of studies have also been performed in Ethiopia (2001) and Nigeria (2011–2013) giving evidence to the presence of PPRV antigen in camels (8, 9) and supporting that camels could be infected by PPRV. Even though there was an outbreak of PPR in camels in mid-July 2013 in Iran with devastating clinical signs caused by lineage IV PPRV (15), experimental infection showed that camels infected with a virulent PPRV strain (lineage IV) did not develop any clinical symptoms of the disease, and no virus was detected in secretions although seroconversion was observed after 14 days of post-infection (73).

#### PPR Infection in Typical Host or Small Ruminants

There were many reports about small wild ruminant species infected by PPRV in the United Arab Emirates (21). On the other hand, white-tail deer challenged with PPRV exhibited clinical signs similar to those in goat (29). Abundant reports of natural infection of PPR disease in gazelles, ibexes, bharals, wild goats (*Capra aegagrus*), wild sheep (*Ovis orientalis*) have also been documented (7, 14, 20, 22, 23, 26, 31). Additionally, Barbary sheep (*Ammotragus lervia*) and Afghan Markhor goat (*Capra falconeri*) died from PPRV infection, which belongs to lineage IV (21). Likewise, in Tibet, China, 19 free-living wild Bharals (*Pseudois nayaur*) showed clinical signs similar to PPR including mucopurulent discharge and severe diarrhea in a pasture nearby where other abnormally dead bharals were prevalent (22). Surprisingly, in India, Chowsingha (*T. quadricornis*), a four horned antelope belonging to the subfamily bovinae and family Bovidae was reported to be affected by PPRV lineage IV (74). Such kind of unusual infection in unusual host requires strong surveillance to strengthen the PPR eradication program. Interestingly six Mongolia gazelles (*Procapra gutturosa*) found dead in a pasture were also discovered to be infected by PPRV lineage IV based on the clinical, serological, and molecular evidences. Above all, an outbreak of PPRV lineage II in Hydropotes inermis (water deer), a rare wild ruminant endemic to China has been reported (32). Experimentally infected West African dwarf goats also showed PPRV virulence (75). Due to the limitations of economic conditions in Africa, prevalence distribution and host range of PPRV have not been well-demonstrated. A recent study showed that PPRV antigen and nucleic acid were detected in specimens from free-ranging dorcas gazelles (*Gazella dorcas*) in Sudan using an immunocapture ELISA and RT-PCR assays (24). Phylogenetic analysis showed that PPRV detected in these gazelles belonged to the lineage IV genotype. With the continuous development of free-animal husbandry, there is a greater chance of interaction between free-living wildlife and domestic species, and it is difficult to monitor PPR in the free-ranging wildlife species thereby increasing the risk of interspecies transmission.

#### PPR Infection in Wild Animals and Dogs

In addition to infection of PPRV in the large and the wildlife ruminants as described above, unexpectedly PPRV infection was reported in carnivore animals as well (7, 13, 33, 34). PPRV was reported to be isolated from an Asiatic lion (*Panthera leo persica*), and multigene sequencing analysis showed that the strain belonged to lineage IV and was closer to the Indian strains (33). Meanwhile, PPRV genome was recently detected from nasal swabs (3/12) of dogs with CDV, and the sequencing results showed 99% identity with PPRV (34).

#### PPR Infection in Pigs

Pigs experimentally infected with PPRV lineage II showed characteristic clinical signs of PPR (35). Although the transmission from infected pigs to healthy pigs or from infected pigs to goats is not reported, it might spread from ill-goats to pigs (35). Therefore, pigs are considered as dead-end hosts for PPRV. However, a recent study of a virulent PPRV lineage IV infection in domestic pigs and wild boar showed that PPRV could be transmitted from pigs to goats and pigs and from goats to pigs (36), indicating that pigs could be a possible source of PPRV infection. Therefore, further investigation on the role of suids in the spread of PPRV in field and experimental conditions with different PPRV lineages and strains is very important. Similar studies on natural infection of PPRV in pigs are still missing, while there is no evidence about pigs as a susceptible animals. The experimental infection with PPRV Nigeria75/1 in suckling mice caused clinical signs in 25% of Balb/C and 24% of Cd1, respectively, but not in C57 (37); however, the transmission of virus to mice from susceptible animals has not been reported yet. Recent report on the detection of PPRV RNA in *Culicoides imicola* have indicated that PPRV might be a vector borne disease (38).

### Mechanisms for Peste Des Petits Ruminants Virus Interspecies Infection

**Figure 3:**
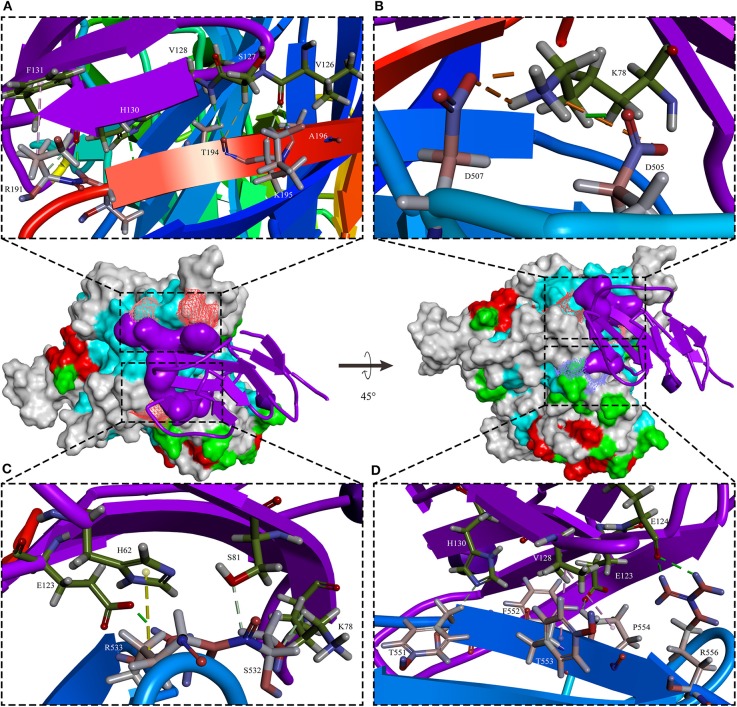
The sSLAM receptor-binding sites with PPRV HNw. The typical β-sandwich structure with BED and AGFCC′ β-sheets of the sheep SLAM (purple) bound to the β4–β6 hydrophobic groove governs in PPRV HNw head domain six-bladed β-propeller head (rainbow colors). In addition to the presence of a large number of hydrogen bonding interactions, D507 on PPRV HNw made an intermolecular salt bridge with K78 on sSLAM-V and also had Pi interactions with PPRV HN residues R191, R533, Y553, and SLAM residues F132, H63, K129. **(A–D)** showed four small segments of the binding interface in PPRV HNw-shSLAM complex.

PPRV has a broad host range in comparison to the closely related MV and RPV, but it does occur in CDV and CeMV which have been reported to be able to cross the species barriers to infect a wide range of hosts (76, 77). For example, CDV in non-dog hosts has been reported in almost all continents. However, the mechanisms beyond this interspecies infection remain unclear. Like other members of morbilliviruses, PPRV glycoprotein HN, which interacts with the cellular receptors, is necessary for virus attachment. It has been demonstrated that signaling lymphocyte-activation molecule (CD150/SLAM) and poliovirus receptor-like protein 4 (Nectin-4/PVRL4) are two major cellular receptors required for morbilliviruses including PPRV to enter the cells (78). In addition, recently microRNA-218 has been reported to affect PPRV replication by regulating SLAM receptor expression facilitated by HN protein of PPRV in PBMC cells (79). Amino acid variations in the CDV protein which binds cellular receptor may play an important role in species specificity (80). From the evolutionary trees, the evolution of H protein of morbilliviruses appears to be strongly associated with the evolution of the cellular receptor (Figure 2A), especially in Nectin-4. The phylogenetic tree of SLAM and Nectin-4 shows that seals (*P. largha*) and walrus (*O.r.divergens*) were located on an evolutionary branch, which indicated that the walrus may be infected with PDV (Figure 2B). However, the fact linked with viruses and hosts which caused the divergence or convergence in the phylogenetic trees remained unclear. Two studies showed that the rate of variation of PPRV HN gene is higher than that of the MV H gene (66, 81). In addition, the variation of PPRVHN gene is faster than that of the genome, which may be the result of the ability of PPRV HN to adapt to large host immune pressure. We have previously reported several positively selective sites on PPRV HN (68), while no positively selective site was found in other morbilliviruses including MV. This indicates that PPRV HN protein does not have much higher stability in comparison with other morbilliviruses, which would have a positive impact on a wide range of host adaptation. On the other hand, a recent phylogenomic analysis based on partial N gene of PPRV with limited sequence data showed a close relationship between PPRV strains recovered from wild and unusual hosts of the same geographical region. From the findings that camel-originated strains from Pakistan clustered close enough to those of domestic origin PPRV reported previously from Pakistan and China (82), it can be inferred that host factors may have a critical role in susceptibility to PPRV infection. On the other hand, SLAM receptor was thought to be related to interspecies infection with the morbilliviruses (64, 83). A study reported that PPRV HN has a high affinity to sheep SLAM based on the analysis of interaction energy and interaction surface contact area (68). When the 188–606 amino acids of PPRV HN were aligned with those of MV H, 41.9% sequence identity and 61.1% sequence similarity were observed. Likewise, the 32–140 amino acids of sheep SLAM (sSLAM)-mice SLAM (mSLAM) and hSLAM (human SLAM)-mSLAM showed 63.7 and 87.3% sequence identity and 79.4 and 93.1% sequence similarity, respectively. This high homology among them promoted the use of the crystal structure of the MV H-SLAM complex (83) as a model to analyze the interaction of PPRV HN protein with SLAM receptor. Simulation of PPRV HN-sSLAM complex showed the presence of a large number of hydrogen bonding interactions in the interface of the complex; D507 on PPRV HN made an intermolecular salt bridge with K78 on sSLAM V domain and also had Pi interactions with PPRV HN residues R191, R533, Y553, and SLAM residues F132, H63, K129 (Figure 3).

**Figure 4:**
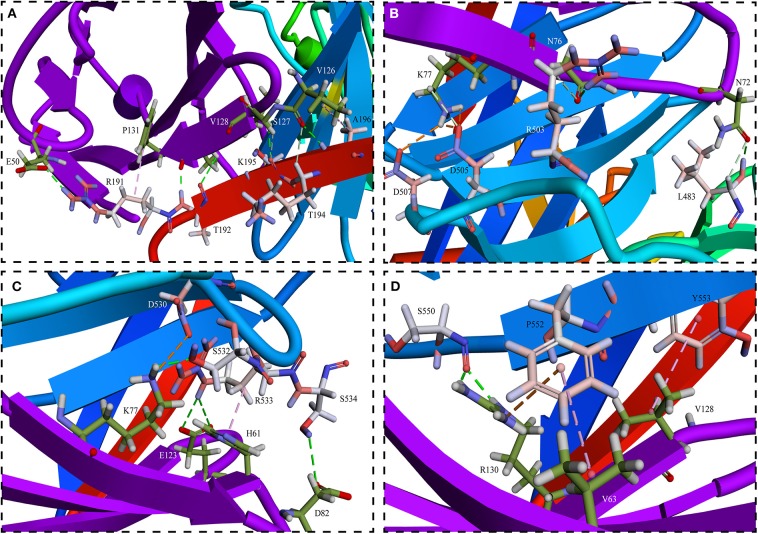
Detailed views of **(A–D)** in the interface between PPRV HNw and hSLAM. **(A)** The interface of PPRV HNw–hSLAM complex, the successional interaction comprised of β-sheet R191-K196 on the PPRV HNw β6 sheets and K126-P131 on the hSLAM G β-sheets made the complex in a more stable state. And R191 of PPRV HNw made the intermolecular Pi interaction with P131 of hSLAM, which might play an important role in stabilizing the PPRV HNw-hSLAM complex. **(B)** The residues D505 and D507 on PPRV HNw made the strong salt bridge with K77 on hSLAM might also play an important role in stabilizing the PPRV HNw–hSLAM complex. **(C)** Key residues D530 and R533 of PPRV HNw made hydrogen bonds and Pi interaction with the residues of hSLAM. **(D)** The strong Pi interaction comprised of residues F552 on PPRV HNw and K64 and R131 on hSLAM. Other residues L483, R503, S532, S534, S550 also made a large number of hydrogen bonds with the SLAM.

Phylogenetic analysis of the receptors showed that the receptors for human and mouse were the most distant compared to goats (Figure 3). Balb/C and Cd1 mice could be experimentally infected by PPRV Nigeria 75/1 strain, but the role of SLAM receptor during PPRV infection in these species is still unknown. If the closest SLAM for evolution has a higher affinity with the PPRV HN, it would be believed that the receptors in other species have also an affinity with the PPRV-HN. Simulation analysis showed that the major binding interface of PPRV HN-hSLAM complex is very similar to that of MV H-SLAM complex (unpublished data). Therefore, we explored the probability of PPRV interspecies infection events from host receptor binding properties using the most distantly related natural hosts of PPRV. Residues I194, D505, D507, D530, R533, F552, and P554 in MV H have been identified as important binding sites for SLAM by multiple mutagenesis studies (84, 85). A comparison of the key amino acids located at the interface of PPRV HN-hSLAM complex showed that residues D505, D507, D530, R533, and F552 were highly conserved in PPRV HN and MV-H proteins (Figure 4). A mutagenesis study confirmed that the β4–β5 hydrophobic groove of H protein head domain is the binding site for both CD46 and Nectin-4, but this hydrophobic groove was not a key binding site for the viral entry through SLAM, which interacts functionally with the propeller blades β5–β6 in MV H (86). Furthermore, this study revealed that hSLAM interacts with an intermolecular β-sheet of PPRV-HN head domain β5–β6 involving the key β64s P191–R195. The mutagenesis and crystal structure study showed that the important residue H61 of mSLAM made a Pi interaction with R533 of MV H, and the contact with E123 of SLAM seemed to stabilize the Pi interaction. Our simulation study shows that R533 of PPRV-HN made a Pi interaction and two hydrogen bonds interact with H61 and E123, respectively. In addition, the crystal structure also showed that R130 had stacking interaction with residue F552 of MV H, while in addition to the formation of Pi interaction, R130 also forms a large number of hydrogen bonds with Y192 and S550 of PPRV-HNw. Considering the receptor, many residues (E50, I60, H61, V63, N77, V82, E123, S127, V128, and F131) are highly conserved in various species. This indicated an affinity of PPRV HN and SLAM of different species by comparing the interaction energy and interaction surface contact area which might help in prediction of the potentiality of the virus to expand to more new hosts species excluding humans.

Besides these factors, Herzog et al. have suggested a relationship between pastoral production and PPRV infection. This study has shown that the animals raised in agropastoral (AP) has seroprevalence rate of 5.8%, whereas 30.7% sero prevalence was observed in pastoral (P) villages in northern Tanzania indicating that even the management system affects the PPRV infection (87). Since unrestricted movements of small ruminants also give rise to massive spread of PPRV as demonstrated in Pakistan by the introduction of disease from infected sheep and goats of Sindh Province (north-west) to Punjab province (central) of Pakistan, health clearance certificate before movement of animals should be emphasized (88).

## Conclusion

Even though effectively attenuated vaccines have been widely used to protect sheep and goats against PPRV, it remains endemic in several parts of the world. Previous studies suggested that the continual spread of PPRV could be related to the emergence of new PPRV strains and lapses in regulatory control (89), but over the last decades the host range of PPRV has been continuously expanding, and the interspecies infection was reported to occur in many non-natural hosts, which may result in multiple ruminant species reservoirs, leading to the silent spread of PPRV due to interaction of livestock with free-living wildlife. Several reports had raised concerns that global PPRV eradication by 2030 may not be achieved as successfully as RPV (52, 90, 91) partially due to the wide host range of PPRV (92) which should be taken into consideration when the global eradication of PPR is implemented. It was observed that in rural areas where small ruminants and cattle coexist and graze together on the same pasture, cross-species transmission of PPRV from small ruminants to cattle is likely to take place frequently (17). Therefore, understanding the importance and roles of these animals in PPRV transmission and evolution might be crucial for effective eradication of this disease. Particularly, PPRV infection in swine and carnivore requires further molecular and cell biology studies on the ability of PPRV to adapt to multiple host. A phylogenetic tree based on the viral HN and the viral receptors (SLAM and Nectin-4) suggested a stable coevolutionary relationship between PPRV and its hosts. Detailed information about the incidence of natural infection, clinical signs and pathology, and confirmation of the virus in these species as well as their role in the epidemiology of PPRV is still lacking. Additionally, limited viral sequences from these animals are available. Therefore, more viral sequences should be generated representing wild and unusual hosts. Besides comparative sequence analysis, analysis on a range of host factors which may be associated with susceptibility of novel host to PPRV infection should be emphasized. Full elucidation of underlying mechanisms on PPRV evolution in relation to interspecies infection would make a positive contribution to successful global PPRV eradication by 2030.

## Author Contributions

YD and ZL design of the study, organized the database, and wrote the first draft of the manuscript. MP and RZ organized the part of database and references. YL and ZZ contributed conception and wrote sections of the manuscript. All authors contributed to manuscript revision, read, and approved the submitted version.

### Conflict of Interest

The authors declare that the research was conducted in the absence of any commercial or financial relationships that could be construed as a potential conflict of interest.
